# A Moderated Mediation Analysis of the Effects of the COVID-19 Pandemic on Well-Being and Sport Readiness of Italian Team Sports Players: The Role of Perceived Safety of the Training Environment

**DOI:** 10.3390/ijerph19052764

**Published:** 2022-02-27

**Authors:** Francesca Vitali, Elisa Bisagno, Marinella Coco, Alessia Cadamuro, Nelson Mauro Maldonato, Donatella Di Corrado

**Affiliations:** 1 Department of Neurosciences, Biomedicine and Movement Sciences, University of Verona, 37129 Verona, Italy; francesca.vitali@univr.it; 2 Department of Law, University of Modena and Reggio Emilia, 42121 Modena, Italy; elisa.bisagno@unimore.it; 3 Department of Biomedical and Biotechnological Sciences, University of Catania, 95123 Catania, Italy; marinella.coco@gmail.com; 4 Department of Biomedical, Metabolic and Neural Sciences, University of Modena and Reggio Emilia, 42121 Modena, Italy; alessia.cadamuro@unimore.it; 5 Department of Neuroscience and Reproductive and Odontostomatological Sciences, University of Naples Federico II, 80138 Naples, Italy; nelsonmauro.maldonato@unina.it; 6 Department of Sport Sciences, Kore University, Cittadella Universitaria, 94100 Enna, Italy

**Keywords:** perceived COVID-19 risk, perceived safety of the training environment, team sports, well-being, sport readiness

## Abstract

Background: The protective restrictions (e.g., lockdowns, quarantines, social and physical distancing) consequent to the global pandemic caused by COVID-19 posited new challenges to athletes practicing competitive team sports. This study aimed to gain an understanding of the impact of the ongoing COVID-19 pandemic on the well-being and sports readiness to train and to compete of competitive female and male athletes practicing outdoor (i.e., rugby, soccer) and indoor (i.e., volleyball, basketball) team sports who were active during the Italian first and second waves of COVID-19. Methods: An online survey assessing demographic characteristics, perceived safety of the training environment, COVID-19 risk, fear of COVID-19, well-being, and sport (training and competition) readiness was administered to 619 team sports players. We examined differences by gender, previous COVID-19 experience, and team sport setting (i.e., outdoor vs. indoor). A moderated mediation analysis was conducted to assess the impact of perceived COVID-19 risk and fear of COVID-19 on athletes’ well-being and sports readiness, using perceived safety of the training environment as a mediator and gender and sport setting as moderators. Results: Indoor team sports and female athletes showed higher perceived COVID-19 risk, while athletes with no-COVID-19 experience reported higher fear of COVID-19. Perceived COVID-19 risk (directly and via perceived safety of the training environment) and fear of COVID-19 were negatively associated with athletes’ well-being and sports readiness. Conclusions: This study highlighted an understanding of the psychological implications of the COVID-19 pandemic, emphasizing the role of the perceived safety of the training environment on athletes’ well-being and sports readiness. Future studies may advance safety-based interventions to promote well-being and a safer return to sport.

## 1. Introduction

In late December 2019, cases of life-threatening pneumonia were attested in Wuhan, China. A novel coronavirus (COVID-19) was documented as the cause of infection, able to penetrate the central nervous system via an olfactory neural pathway. The number of testified cases rapidly increased, confirming this virus as a public health emergency of international concern. The transmission of COVID-19 occurs principally through droplets and strict contact [1]. The immediate line of defense against this pandemic was governments’ protective limitations, including full lockdowns of nations, regions, and municipalities, deletion of travels, and restrictions in social congregations (e.g., restaurants, concerts, movie houses, fitness centers, sports events), the closing of educational institutions, and suppression of sports tournaments [2].

Starting from the first prolonged quarantine that began on 11 March 2020 and ended on 3 May 2020, due to the COVID-19 pandemic in Italy, the daily habits of individuals have deeply changed, requiring them to practice “social and physical distancing”. Home confinement, while being a safety measure to avoid human-to-human transmission of the virus, had unintended negative consequences, which damagingly impacted individual well-being. Rossi et al. [3] revealed symptoms of anxiety, depression, self-reported stress, and sleep disorders in response to the pandemic within the general population. Higher rates of anxiety and depression were recorded in women, students, and younger adults. Delmastro and Zamariola [4] assessed 18,147 Italian individuals and found high levels of post-traumatic stress symptoms (37.14%), depression (17.3%), anxiety (20.8%), sleep disorders (7.3%), perceived stress (21.9%), with female respondents expressing higher impact on their mental-ill health. On top of this, while recent studies [5,6,7] have underlined a strong health rationale for continuing PA while taking precautions, another element that substantially contributed to disrupting people’s well-being during the first COVID-19 wave was the fewer possibilities to practice physical activity (PA) and sports, with an increase in sedentary behaviors, and screen time [8], as well as significant disruption in the training possibilities of competitive and elite athletes.

Many studies investigated the main risk factors (i.e., age, gender, expertise, type of sport) for a detrimental impact of the COVID-19 pandemic in the specific population of athletes. Pillay et al. [9] evaluated elite and semi-elite South African athletes during the final phase (last week of April 2020) of their domestic lockdown. The results showed an increase in sleep disorders, depression, and overweight.

Conde et al. [10] examined the effects of the COVID-19 pandemic on sports practice, life quality, and emotional states in 130 elite Spanish athletes and found three risky profiles: team players, student-athletes, and female athletes. The first profile experienced a longer rest days period compared to individual sports athletes during the lockdown and greater distress due to social distancing. The student-athletes pursuing academic studies and sports careers are framed in the so-called dual career [11], where a high level of effort and many hours of dedication are required to achieve such a conciliation. During the Spanish lockdown, they also spent more time on rest-related activities (days and hours) compared to the pre-lockdown normality. Differences were found between genders in health perception, with female elite athletes scoring significantly lower than their male counterparts compared to their perception in pre-lockdown. In relation to the performance perception, both genders showed a significant decrease in the post- compared to the pre-lockdown period (with a higher decrease expressed, again, by female elite athletes).

Ruffault et al. [12] explored differences in anxiety and motivation to return to sport agreeing to gender, expertise, and training status before and during the lockdown in 759 French competitive athletes. Group assessments revealed higher levels of anxiety for female athletes, younger players, athletes competing at the highest level, and athletes without a training program during the French lockdown. The authors suggested that elite athletes might have suffered from external pressures to return to sport during the lockdown.

More importantly for the purpose of this paper, several studies [13,14] have documented the negative effects of lockdown on Italian athletes who, in addition to the aforementioned problems, also suffered from the sudden interruption of their training sessions and competitions. In Italy, as a protective strategy to reduce the COVID-19 outbreak, the first national lockdown was started on 11 March 2020, and all sports’ training sessions, competitions, and tournaments were interrupted for about a dozen days to preserve the social distancing. This interruption involved both elite and amateur athletes. After this first national lockdown, the vast majority of elite Italian athletes during the first COVID-19 wave restarted to train and, in some cases (e.g., soccer), continued the elite union competition tournaments in the short-term without inducing changes in the epidemiology of sports injuries [15], leaving today some long-term concerns about the physical condition of the football players [16]. On the contrary, the national elite female and male rugby, volleyball, and basketball unions tournaments were suspended together with the respectively grassroots sports training sessions, competitions, and tournaments.

Di Fronso et al. [13] assessed 1132 competitive athletes’ perceived stress and functional/dysfunctional psychobiosocial states during the very beginning of the COVID-19 crisis, comparing these evaluation scores with data collected before the pandemic. Differences by gender, type of sport (i.e., individual vs. team sport), and competitive level (expert vs novice athletes) were observed. Coherently with the general Italian population, female athletes showed higher perceived stress and dysfunctional psychobiosocial states scores than male athletes, as well as lower functional psychobiosocial states scores. Expert athletes reported lower perceived stress and higher functional psychobiosocial states scores than novice athletes. No significant differences emerged between athletes practicing individual or team sports.

Di Cagno et al. [14] examined differences by gender, type of sport (i.e., individual vs. team), and competitive level (elite vs. amateur) in 1508 self-selected Italian adult athletes. Findings showed significant differences between genders for perceived stress and avoidance behavior, with higher scores in female athletes. In considering individual and team sports, significant differences were found in distress, and hyperarousal, with higher results in athletes involved in individual sports.

In May 2020, after two months of national lockdown, it was permitted to resume both elite and grassroots team sports competitions and tournaments. However, various restrictions such as physical distancing in sports facilities, clubs, and fields, the inability to use locker-rooms, and showers, the use of face masks, gloves, and alcohol-based hand sanitizers, together with the need to limit shared materials, and regularly check for COVID-19 positive athletes have strongly conditioned this rebooting phase.

Guicciardi and Pazzona [17] have conducted a web-based survey at the initial phase of the PA and sports’ rebooting phase after the first lockdown in Italy (beginning of May 2020) to examine how individuals responded to the new rules when practicing PA and sports, and which effects could have rebooted. Participants were amateur athletes practicing team (i.e., soccer, basketball) and individual (i.e., running, bodybuilding) sports who completed questionnaires assessing sleep quality, regulatory self-efficacy, optimism, mood states, and mental toughness. In outdoor PA and sports, younger women showed less regulatory self-efficacy when compared to older women, while younger men manifested higher regulatory self-efficacy than older men. Moreover, younger participants showed more sleep disorders, confusion, depression, anger, less vigor, and mental toughness than older participants.

In October 2020, the Italian government imposed a second national lockdown that stopped for a second time the sports clubs’ routine. Training sessions and competitions tournaments were canceled again, as well as travels to national meetings and international matches. Added stressors to athletes were career disruption also linked to postponement of major sports events (e.g., World Rugby Six Nations Tournament, Summer Olympic and Paralympic Games Tokyo 2020), vagueness regarding calendars, and rules of major competitions, and uncertainty on qualifying tournaments.

In this frame, both training sessions and matches were subject to several procrastinations, momentary stops, and numerous restrictions (e.g., absence of supporters during matches). In this sense, the rebooting phase in sports is still requiring athletes to adapt old habits to new rules but also to face potential new fears related to the perceived risk of COVID-19 contagion. It is also worth noting that, while athletes competing in individual sports might have experienced higher distress during the lockdown phase due to lesser chances to express and share feelings [17], on the other hand, athletes practicing team sports have been declared at higher risk of contracting the virus while training and competing [18], which is an important element to take into account when designing the rebooting phase in sports during the COVID-19 pandemic. Different and more pressing challenges might be presented to team sports athletes during this phase, and, in this sense, a key role might be played by the perceived safety of the sports environment, defined as the degree to which a person can manage psychological and physical pressures from the environment [19]. According to the National Research Council and Institute of Medicine [20], physical and psychological safety of the environment is one of the key features that can facilitate positive growth and well-being in youth and reduce the perceived COVID-19 risk and fear. Sport is an inherently social activity. Interactions with teammates, coaches, operational staff members, officials, managers, and broader sports communities represent an integral component of the sports experience. Within the specific context of the COVID-19 pandemic, it is these social interactions that are being directly challenged, also in reason of high levels of physical and psychological uncertainty, and this appears true not only for youth athletes [21].

Indeed, several international organizations established protective measures to ensure the actual and perceived safety of athletes [22]. In addition, it is known that in many work environments (e.g., hospital settings) [23], the perceived safety of the environment during the COVID-19 pandemic greatly impacted personal well-being.

According to these considerations, it would not be surprising if the perceived risk and fear of contracting COVID-19 impacted the well-being and sports readiness of athletes to train and to compete, and if the perceived safety of the training environment played a relevant role in determining these associations. These considerations are even more pertinent for team sports, where specifically, relationships and contacts with teammates are certainly higher if compared to those of individual sports athletes.

## 2. Aim of the Study

This study aimed to gain an understanding of the impact of the COVID-19 pandemic on the well-being and sport (training and competition) readiness of competitive female and male athletes practicing outdoor and indoor team sports who were active during the Italian first and second waves of COVID-19, based on both dispositional, and environmental variables. Specifically, we aimed to analyze differences in the athletes’ perceived safety of the training environment, perceived COVID-19 risk, fear of COVID-19, well-being, and sports readiness to train and to compete based on their gender, previous COVID-19 experience, and sport setting (outdoor vs. indoor). Moreover, we aimed to analyze the effects of perceived COVID-19 risk and fear of COVID-19 on well-being and sport (training and competition) readiness to better understand the interplay among these variables in determining athletes’ well-being and sports readiness to train and to compete. To do so, we chose to examine team sports that differ by the type of setting and environment, comparing outdoor (i.e., rugby, soccer) and indoor (i.e., volleyball, basketball) team sports. In line with the literature, we predicted that:

**Hypothesis** **1** **(H1).** *Perceived COVID-19 risk and perceived COVID-19 fear will be negatively associated with well-being and positively associated with reduced sports readiness to train (training readiness bias) and to compete (competition readiness bias).*

**Hypothesis** **2** **(H2).** *Perceived safety of the training environment will mediate these associations.*

**Hypothesis** **3** **(H3).** *Sport setting (indoor vs. outdoor) will moderate these associations, with stronger associations for indoor(i.e., volleyball, basketball) team sports*.

**Hypothesis** **4** **(H4).** *Gender (female vs. male) will moderate these associations, hypothesizing stronger associations for female players.*

## 3. Materials and Methods

### 3.1. Eligibility Criteria

Eligibility criteria to participate in this study were the following: (1) being female and male team sports athletes; (2) being aged 18 years or more; (3) being competitive players; and (4) being an active team sports athlete during the Italian first and second waves of COVID-19.

### 3.2. Procedure

Data were collected through an anonymous online survey (Google Forms, Google, Mountain View, California, CA, USA) administered from 20 March to 31 May 2021. The time required to complete the survey was approximately 15 minutes. Participants were recruited thanks to the support of sports federations and clubs, involved using social media advertisements (i.e., Facebook, LinkedIn, Twitter, WhatsApp profiles, and accounts of the first, second, and last authors of this paper). All participants signed a free written consent to participate after receiving a full description of the protocol of the study and their rights to anonymity. All the procedures were conducted in accordance with the ethics of the Declaration of Helsinki, and the University Enna Kore Internal Review Board for psychological research (UKE-IRBPSY-02.21.03) gave ethical permission.

### 3.3. Measures

The online questionnaire was administered to participants through a link (i.e., https://forms.gle/CtuTGcn9toycKCfV8, access on 17 June 2021). The survey consisted of two parts: the first one collected sociodemographic data (i.e., gender, age, sport, level of expertise, years of sport experience, deliberate practice), and two control variables: the duration of stop from sports practice due to COVID-19 measured in number of months, and direct or indirect experience of COVID-19 (i.e., personal COVID-19 contagion or COVID-19 cases in teammates, coaches, and operational staff, or not). The second part of the survey included several scales detailed as follows.

#### 3.3.1. Fear of COVID-19 Scale

An adapted version of the Fear of Coronavirus-19 Scale (FCV-19S) [24] was used to assess the emotional fear reactions towards the ongoing COVID-19 pandemic. The scale consists of seven items (e.g., “I am most afraid of the COVID-19”) measured on a Likert-type scale ranging from 1 (strongly disagree) to 5 (strongly agree). One item was changed from “I cannot sleep because I’m worried about getting COVID-19” to “I cannot train because I’m worried about getting COVID-19”. The total score ranges between 7 and 35, with a higher sum score indicating higher fear of COVID-19. In our study, the FCV-19S showed good internal consistency in terms of Cronbach’s alpha (α = 0.89).

#### 3.3.2. Perceived Safety of the Training Environment

We developed a 5-items ad hoc questionnaire (see Supplementary Materials) measured on a Likert-type scale ranging from 1 (strongly disagree) to 5 (strongly agree) to assess the perceived safety of the training environment for athletes. Some examples of items are: “My Club takes the necessary safety measures to minimize the chances of COVID-19 contagion”, and “I believe that my teammates respect the safety measures”. The score is calculated as the mean of the five items, and it can range between 1 and 5, with a higher mean indicating higher perceived safety. In our study, the internal consistency (Cronbach’s alpha) was 0.88.

#### 3.3.3. Perceived COVID-19 Risk Scale

We measured the perceived risk of contracting the COVID-19 through the Perceived Coronavirus Risk scale (PRCS), adapted by Kanovsky and Halamová [25] from the Perceived Risk of HIV (PRHIV) [26]. The scale consists of 8 items measured on a Likert-type scale ranging from 0 (strongly disagree) to 5 (strongly agree) and assessing the perceived risk of contracting the COVID-19. Items 3 and 4 are reversed. The score is calculated as the mean of the 8 items after reversing items 3 and 4, and it ranges between 0 and 5, with a higher mean indicating more perceived risk of COVID-19. In our study, Cronbach’s alpha was 0.90.

#### 3.3.4. World Health Organization Well-Being Index

The 5-items World Health Organization Well-Being Index (WHO-5) [27] is a short, global rating scale measuring subjective well-being. The WHO-5 comprises 5 positive statements (e.g., “I have felt calm and relaxed”) preceded by the headline “In the past two weeks…”. To properly assess the athletes’ global well-being during the COVID-19 pandemic, we changed it into: “From the beginning of the COVID-19 pandemic in Italy…”. Each of the 5 items is scored from 0 (never) to 5 (always). The score, therefore, ranges from 0 (absence of well-being) to 25 (maximal well-being). In our study, the scale had good internal consistency in terms of Cronbach’s alpha (α = 0.88).

#### 3.3.5. Sport Readiness

We developed a 4-items ad hoc questionnaire measured on a Likert-type scale ranging from 0 (not at all) to 5 (very much) to assess the athletes’ readiness to train (items 1 and 2) and to compete (items 3 and 4), before and after the COVID-19 pandemic. Two scores (training readiness bias and competition readiness bias) were computed as before-after pandemic biases. A higher before-after pandemic bias means lower readiness to train/compete than usual. In our study, Cronbach’s alpha was 0.80.

### 3.4. Statistical Analysis and Sample Size

Data were expressed as means (*M*) ± standard deviations (*SD*) and the range. A 2 × 2 × 2 (outdoor/indoor × gender × COVID-19/no-COVID-19) multivariate analysis of variance (MANOVA) was conducted on the data of the study variables. Follow-up analysis of variance (ANOVA) was then used to define the significant differences. Furthermore, Pearson’s correlation was used to determine the relationships between the selected variables. An a priori power analysis was run with G*Power [28]. This analysis found a multiple regression analysis with 9 predictors (two independent variables, one mediator, and two moderators on both direct and indirect path), an alpha level of 0.05 (two-tailed), a power of 0.80 to detect a small to medium effect size of ρ^2^ = 0.06, required total sample size of *N* = 270. Therefore, a total of *N* = 619 participants were recruited to take part in the research.

We decided to assess the impact of perceived COVID-19 risk and fear of COVID-19 on athletes’ well-being and sports readiness through a moderated mediation analysis, using perceived safety of the training environment as a mediator, and gender and sport setting as moderators (a graphical representation of the model will be plotted in the Results section). In statistics, mediation analysis is used to quantify a causal sequence where an independent variable (in our case, COVID-19 risk) causes a mediating variable (in our case, perceived safety of the training environment) that, in turn, causes one or more dependent variables (in our case, well-being, training readiness bias, and competition readiness bias). In this sense, the mediator explains the relationship between the independent and the dependent variables. A moderator analysis is used to specify whether the relationship between two (or more) variables depends on the value of other variables, namely the moderators (in our case, sport setting and gender). As in our study, moderation and mediation can occur together in a single model, thus causing conditional indirect effects (that is, both the direct and the indirect effects of the independent variable on the dependent variables can depend on the moderator value).

Therefore, a moderated mediation analysis was conducted using Hayes’ [29] PROCESS version 3.5 computational tool for SPSS (Model 10). This tool enables estimation of path coefficients, standard errors, and different indexes of effect size, as well as the significance of the indirect effects obtained through the bootstrapping method with 5000 repetitions, with a confidence interval (CI) of 95% [30]. Statistical significance was set at *p* ≤ 0.05. Statistical analyses were processed using SPSS version 25.0 (IBM, Armonk, NY, USA).

## 4. Results

### 4.1. Participants

Even though *N* = 672 participants enrolled in the online survey, *n* = 53 questionnaires were unfinished and consequently removed from the final dataset. A total of *N* = 619 competitive team sports players (*n* = 284 female and *n* = 335 male) who were active during the Italian first and second waves of COVID-19, aged between 18 and 45 years (*M*_age_ = 24.09; *SD* = 5.90), took part in the study. Based on the athletes’ highest levels of performance, they were classified as the Italian Serie A (i.e., 1st league; *n* = 209), the Italian Serie B (i.e., 2nd league; *n* = 224), and the Italian Serie C (i.e., 3rd league; *n* = 186). Participants belonged from different Italian regions and practiced the following team sports: rugby (M = 116; F = 48), soccer (M = 71; F = 55), volleyball (M = 79; F = 126), and basketball (M = 69; F = 55). All competitive athletes had a minimum of three years of practice experience in their sport. The sports selected were representative of outdoor team sports (i.e., rugby and soccer), and indoor team sports (i.e., volleyball and basketball). We collected the deliberate practice amount (i.e., training sessions per week and hours of training per week) at the moment of the survey. In addition, we measured the duration of the stop from sports practice due to COVID-19 (less than six months vs. more than six months) and COVID-19 direct or indirect experience (i.e., personal COVID-19 contagion or COVID-19 cases in teammates, coaches, and operational staff, or not). The descriptive characteristics of the participants are shown in Table 1.

### 4.2. MANOVA Results and Correlations

Findings revealed significant multivariate effects for outdoor vs. indoor team sports, Wilks λ= 0.978, *F* (6, 606) = 2.267, *p* = 0.003, *η*_p_^2^ = 0.019, power = 0.730, female athletes vs. male athletes, Wilks λ= 0.954, *F* (6, 606) = 4.817, *p* = 0.001, *η*_p_^2^ = 0.046, power = 0.991, and COVID-19 vs. no-COVID-19 experience, Wilks λ= 0.966, *F* (6, 606) = 3.573, *p* = 0.002, *η*_p_^2^ = 0.034, power = 0.954. Means (*M*) and standard deviations (*SD*) of study variables are reported in Table 2.

Follow-up ANOVAs revealed significant univariate differences between outdoor and indoor team sports on perceived COVID-19 risk variable scores, between COVID-19 and no-COVID-19 experience on perceived safety of the training environment and fear of COVID-19 variable scores, and between female athletes and male athletes on COVID-19 risk and well-being variable scores. Specifically, indoor team sports’ athletes reported higher scores on perceived COVID-19 risk, *F* (6, 606) = 7.696, *p* = 0.003, *η*_p_^2^ = 0.021, power = 0.791 (indoor *M* = 2.74; outdoor *M* = 2.45) than outdoor team sports’ athletes. Athletes with no-COVID-19 experience reported higher scores on perceived safety of the training environment, *F* (6, 606) = 8.001, *p* = 0.005, *η*_p_^2^ = 0.013, power = 0.806 (no-COVID-19 *M* = 4.11; COVID-19 *M* = 3.93) and on fear of COVID-19, *F* (6, 606) = 8.834, *p* = 0.003, *η*_p_^2^ = 0.014, power = 0.843 (no-COVID-19 *M* = 1.21; COVID-19 *M* = 0.94) than athletes with COVID-19 experience. Female athletes reported higher scores on perceived COVID-19 risk, *F* (6, 606) = 10.114, *p* = 0.002, *η*_p_^2^ = 0.016, power = 0.888 (women *M* = 2.75; men *M* = 2.49) and lower scores on well-being, *F* (6, 606) = 11.690, *p* = 0.001, *η*_p_^2^ = 0.019, power = 0.927 (women *M* = 2.22; men *M* = 2.50) than male athletes. The relationships among variables were analysed with Pearson’s correlation (Table 3).

As can be observed, the perceived safety of the training environment is negatively associated with perceived COVID-19 risk and with the training and competition readiness biases. Conversely, the perceived safety of the training environment is positively associated with well-being. On the other hand, the fear of COVID-19 and perceived COVID-19 risk are negatively associated with well-being and positively associated with lower-than-usual training and competition readiness. Lastly, reduced sports readiness both to train and to compete is negatively associated with well-being.

### 4.3. Mediation Analysis

**Figure 1:**
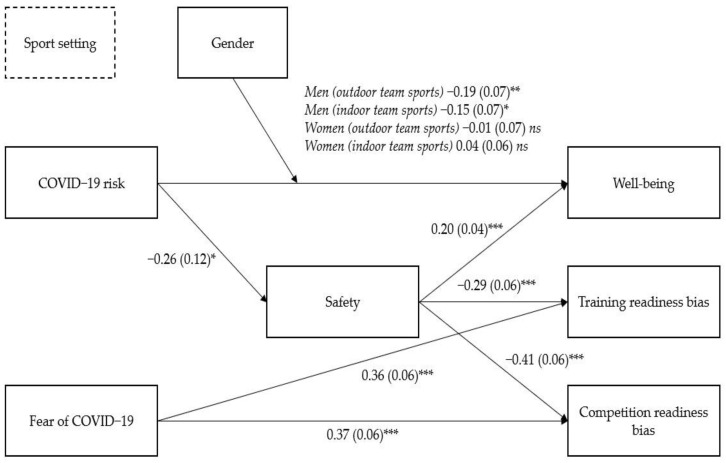
Direct and indirect (via perceived safety of the training environment) effects of perceived COVID-19 risk and fear of COVID-19 on well-being, training readiness bias, and competition readiness bias moderated by gender (but not by sport setting). Note. Unstandardized (standard errors in parentheses) coefficients are reported, * *p* < 0.05. ** *p* < 0.01. *** *p* < 0.001.

Based on the correlation results, moderated mediation analyses were executed. Specifically, we run three moderated mediation models, considering the three dependent variables (i.e., well-being, training readiness bias, and competition readiness bias). In all models, perceived COVID-19 risk and fear of COVID-19 were the independent variables, while perceived safety of the training environment was the mediator, and sport setting and gender were the moderators. In Model 1, the dependent variable was the athletes’ well-being, while in Model 2, the dependent variable was the athletes’ training readiness bias, and in Model 3, it was the athletes’ competition readiness bias. Results are shown in Figure 1 and Table 4, which contains the Bs, indicating the intensity of the effect, and the 95% CIs, indicating the significance of the effect with a 5% probability of error (CIs that do not contain 0 are significant).

In line with H1 and H2, the results showed that perceived COVID-19 risk was negatively associated with well-being both directly, b = −0.38, SE = 0.12, 95% CI [−0.62, −0.14], but also indirectly via decreased perceived safety of the training environment. Moreover, a significant moderation effect emerged in the direct path from the independent variable to the dependent variable. Specifically, contrary to H4, COVID-19 risk was negatively associated to well-being only among male athletes of outdoor (B = −0.19 (0.07), *p* < 0.01, 95% CI [−0.33, −0.06]), and indoor (B = −0.15 (0.07), *p* < 0.05, 95% CI [−0.28, −0.01]) team sports. This relation was nonsignificant for female athletes of outdoor (B = 0.01 (0.07), *p* = 0.91, 95% CI [−0.16, 0.14]) and indoor (B = 0.04 (0.06), *p* = 0.53, 95% CI [−0.08, 0.16]) team sports. The indirect effects are presented in Table 5.

In line with H1 and H2, in Models 2 and 3, perceived COVID-19 risk was positively associated with the outcome variable (training readiness bias for Model 2 and competition readiness bias for Model 3) via lower perceived safety of the training environment (full mediation Models). All the indirect effects are presented in Table 5.

Partially in line with H1 (but not with H2), the fear of COVID-19 was directly (but not indirectly) associated with reduced training, b = 0.36, SE = 0.06, 95% CI [0.25, 0.48], and competition readiness, b = −0.38, SE = 0.12, 95% CI [−0.62, −0.14], but not with well-being, b = −0.06, SE = 0.04, 95% CI [−0.14, 0.02]. Lastly, contrary to H3, the sport setting (outdoor vs. indoor) did not moderate any association.

## 5. Discussion

The COVID-19 pandemic placed massive worries on sports communities due to the increase in many challenges and uncertainties. Since the beginning of the COVID-19 outbreak, the Italian government implemented extraordinary measures to contain and decrease the potential transmission of the virus, including full lockdowns, momentary stops, and numerous restrictions that negatively impacted not only the general population’s life habits but also the sports clubs’ routine. For instance, training sessions, matches, and competitions tournaments were canceled, postponed, or played behind closed doors without the supporters’ presence. Therefore, athletes were asked to interrupt training sessions, competitions, and tournaments, stay home, and take care of their physical fitness training or physical rehabilitation after an injury by themselves or, in a few cases, under the telerehabilitation of a physiotherapist. In the latter case, attending a mixed supervised and self-administered sessions program that was effective in promoting clinical improvements, maintaining positive self-efficacy and well-being, and preventing the worsening of psychological distress [31]. 

Sport should promote the health, well-being, and protect the physical and psychological safety of athletes but also coaches, team and operational staff, officials, managers, volunteers, and other participants, and not unduly increase those individuals’ relative health risk (e.g., the perceived risk of contracting COVID-19) while contributing to rebooting sport, economic recovery, providing entertainment for the public, and leading a responsible restoration of civic life [22].

Our study aimed to analyze differences in competitive Italian team sports athletes’ perceived safety of the training environment, perceived COVID-19 risk, fear of COVID-19, well-being, and sport (training and competition) readiness based on their gender, COVID-19 experience, and team sports’ setting (indoor vs. outdoor), as well as to test a model of the interplay among these variables in determining athletes’ well-being, and sports readiness compared with before the lockdown.

Firstly, findings showed that athletes of indoor team sports and female athletes reported higher scores on perceived COVID-19 risk. These results suggest that athletes practicing indoor team sports, being often in close contact with teammates, coaches, team and operational staff, and other participants, could perceive being at a higher risk of contracting COVID-19 through transmission during training sessions and routine physical activities or matches. Notwithstanding the COVID-19 infection risks of carrying out outdoor vs. indoor sports have not been established yet, it appears likely that the risk of transmission in an indoor sporting environment could be higher. Furthermore, regarding adaptation processes and responses to potentially traumatic events, women usually achieved higher scores on self-perceived stress and mental health disorders [32]. In the specific COVID-19 pandemic situation characterized by a high degree of uncertainness, fear of COVID-19 can be interpreted as incontrollable; therefore, female athletes could experience mood disturbance and feelings of emptiness that may impact mental well-being [3,4].

Athletes with no-COVID-19 experience reported higher scores on the perceived safety of the training environment and fear of COVID-19. Potentially, not having been exposed to the virus yet led them to perceive their training environment as safer; on the other hand, fear of COVID-19 and related uncertain consequences of contagion might be higher for those athletes who have not experienced the COVID-19 illness. The imposed home-confinement strategy, the inability to maintain a normal team routine, and the safety measures (such as avoiding gatherings, impossibility to access locker-rooms and showers, and use of personal protective equipment) may have heightened the fear of COVID-19 contagion and personal insecurity.

This interpretation was supported in our study by correlational data, which revealed that perceived safety of the training environment was significantly and negatively associated with perceived COVID-19 risk and training and competition readiness biases (indicating lower readiness than usual). Conversely, it was positively associated with well-being. On the other hand, the perceived COVID-19 risk and fear of COVID-19 were negatively associated with well-being and positively associated with lower-than-usual training and competition readiness. Lastly, reduced sports readiness was negatively associated with well-being.

As the ongoing COVID-19 pandemic runs its course, worries persist regarding PA and sports safety, resumption of the sporting activities, and how to manage infected athletes. Developing and assuring a safe environment must be a dominant aim in the present and immediate future to ensure athletes are not exposed to the COVID-19 transmission. By creating and maintaining a feeling of safety in the sports environments, federations and clubs can promote exploration, adaptation, creativity, and a sense of well-being not only for youth athletes [21] but also for adult athletes.

This consideration is in line with the last investigation of our study, referred to the moderated mediation models, which almost completely confirmed the initial hypotheses. The perceived COVID-19 risk was negatively associated with well-being directly and indirectly via decreased perceived safety of the training environment. Moreover, the perceived COVID-19 risk was positively associated with the training readiness bias and competition readiness bias via lower perceived safety of the training environment. These findings are in line with recent studies that revealed how uncertainties and fears regarding COVID-19 could negatively influence psychological well-being [33,34].

Partially in line with H1, the fear of COVID-19 is directly (but not indirectly) associated with reduced training and competition readiness, but not with well-being. This might suggest that contrary to perceived risk, the fear of COVID-19 contagion is more based on individual rather than environmental aspects, as suggested by a recent review [35].

Contrary to H3, a moderation effect of the sport setting (outdoor vs. indoor) did not emerge. Lastly, a moderating effect of gender emerged on the direct association between perceived COVID-19 risk and well-being; despite women in the general population experienced more perceived risk and discomfort in general, contrary to H4, the association only occurred among male athletes of outdoor and indoor sports and not for female counterparts.

A possible explanation may be related to the different athletes’ investment in their athletic role according to gender, where male athletes show a higher investment if compared with female athletes [36]. Indeed, previous studies evidenced a higher identity in male athletes than in their female colleagues [37,38]. Moving from the assumption that athletic identity could have been higher within the male athletes’ subsample of our study, a possible interpretation of this apparently incoherent result might be that, for male athletes, the COVID-19 perceived risk within the training and sports environments could be more impactful on overall well-being due to a higher worry to having to stop training and competing if exposed to the virus. This represents a limitation of our study that did not take into account athletic identity as a variable, and, for this reason, our interpretation in this sense represents only a speculation based on previous literature. Future studies may consider the present model and analyze the role of athletic identity within it.

In conclusion, the mediation but not the moderation results confirmed almost all research hypotheses. Specifically, mediation analyses highlighted the role of the perceived safety of the training environment in determining the risk of COVID-19 contagion perceived by athletes, which had a strong impact on their well-being, and reduced sports readiness. Moreover, fear of COVID-19 showed an impact on reducing sports readiness, which, however, is not mediated by the perceived safety of the training environment.

In elite professional sports contexts, a safe resumption of training sessions and competitions in a COVID-19 environment will be a complex process requiring a thorough risk assessment. To reduce not only the transmission of COVID-19 but also to maintain a positive perception of the safety of the training environment, awareness-raising, and education of athletes about COVID-19 risk mitigation strategies and setting expectations for the required behaviors before recommencing activities is crucial [39].

The findings of our study contribute to the current research, highlighting an understanding of the psychological implications of the COVID-19 pandemic and emphasizing the role of the perceived safety of the training environment on team sports athletes’ well-being and sports readiness. This is particularly interesting from an applied perspective because it offers sports stakeholders, such federations and sports clubs, important information on how to facilitate athletes’ rebooting of training sessions and competitions, as well as fostering their personal well-being, by providing them with clear, feasible safety measures to face and prevent COVID-19 contagion.

### Limitations and Perspective

Notwithstanding the promising results, as already mentioned above, this study has some limitations. First, the use of psychometric validated scales—in place of ad hoc questions—to measure the safety of the training environment or sports readiness to train and to compete could give more opportunities for valid data analysis. Second, the opportunity of selection bias due to the online survey should be regarded. A third limitation is that in this study we evaluated well-being without having the same pre-lockdown measure on the sample: having had it would have allowed us to evaluate the effect of the COVID-19 pandemic on this aspect.

Furthermore, future studies should explore beyond the limitations of the present paper and investigate how the same dimensions interact with athletic identity and relate to athletes’ well-being.

## 6. Conclusions

COVID-19 has had devastating effects on communities globally, leading to significant restrictions on daily civic life as well as on sport. Consequently, it is important to recognize the psychological influence of the COVID-19 pandemic to better prepare researchers to look at and assess the psychosocial repercussions not only on the general population but also on the athletic one. Future research should advance safety-based interventions to promote well-being and a safer return to sport. The health and well-being of athletes and other personnel (e.g., coaches, team and operational staff, officials, managers, volunteers) must be an overriding priority for governmental and sports stakeholders’ organizations.

## Figures and Tables

**Table 1:** Descriptive statistics of the sample.

	Age M ± SD (Range)	Training Sessions per Week M ± SD (Range)	Hrs of Training per Week M ± SD (Range)	Duration of Stop from Sport Practice Due to COVID-19		COVID-19 Direct or Indirect Experience		Level of Performance (i.e., Italian Serie)		
				<6 Months	>6 Months	Yes	No	A	B	C
Rugby (*n* = 164)	25.6 ± 6.3 (18–45)	3.6 ± 1.4 (2–9)	2 ± 0.4 (2–4)	110	54	126	38	82	60	22
Soccer (*n* = 126)	23.8 ± 5.4 (18–45)	4.1 ± 1.3 (2–9)	2.3 ± 0.8 (2–8)	98	28	86	40	33	41	52
Volleyball (*n* = 205)	23.3 ± 5.6 (18–45)	4 ± 1.8 (2–10)	2.3 ± 0.5 (2–4)	138	67	126	79	46	65	94
Basketball (*n* = 124)	23.5 ± 5.9 (18–45)	4.4 ± 1.9 (2–10)	2.1 ± 0.3 (2–3)	73	51	89	35	48	58	18

**Table 2:** Means and standard deviations for the study variables.

Variables		Female Athletes				Male Athletes			
		COVID-19		No-COVID-19		COVID-19		No-COVID-19	
		Outdoor *n* = 77	Indoor *n* = 122	Outdoor *n* = 26	Indoor *n* = 59	Outdoor *n* = 135	Indoor *n* = 93	Outdoor *n* = 52	Indoor *n* = 55
Safety	M	3.87	3.97	4.45	4.03	3.95	3.92	4.07	4.09
	SD	0.97	0.82	0.64	0.75	0.98	1.06	0.93	00.78
Fear of COVID-19	M	0.91	0.98	1.21	1.36	0.81	1.09	1.02	1.24
	SD	1	0.89	1.09	1.26	0.88	0.92	1.03	0.92
COVID-19 risk	M	2.43	2.89	2.81	2.85	2.51	2.56	2.18	2.60
	SD	0.98	1.04	0.89	1.08	0.84	0.98	0.99	0.90
Well-being	M	2.16	2.26	2.28	2.20	2.51	2.40	2.44	2.70
	SD	0.97	0.88	1.01	1.03	0.97	0.89	1.01	0.77
Training readiness bias	M	1.13	0.89	0.85	0.76	0.62	0.98	0.87	0.56
	SD	1.38	1.43	1.54	1.48	1.13	1.29	1.45	1.15
Competition readiness bias	M	1.16	1.28	1.23	1.34	0.89	1.15	0.94	0.73
	SD	1.52	1.59	1.53	1.46	1.39	1.48	1.61	1.17

**Table 3:** Pearson’s correlation coefficients.

Variables	1	2	3	4	5	6
1.Safety	1					
2.Fear of COVID-19	−0.045	1				
3.COVID-19 risk	−0.152 ***	0.468 ***	1			
4.Well-being	0.210 ***	−0.111 **	−0.148 ***	1		
5.Training readiness bias	−0.213 ***	0.289 ***	0.189 ***	−0.259 ***	1	
6.Competition readiness bias	−0.270 ***	0.286 ***	0.209 ***	−0.228 ***	0.672 ***	1

Note. ** *p* < 0.01. *** *p* < 0.001.

**Table 4:** Results of moderated mediation analyses *(N* = 619). Unstandardized coefficients (B) (standard errors in parentheses) are reported.

Predictors	Dependent Variable					
	Model 1, 2, and 3: Safety					
	B			95% CI		
COVID-19 risk (a)	−0.26 (0.12) *			[−0.504, −0.021]		
Fear of COVID-19 (b)	0.03 (0.04)			[−0.050, 0.115]		
Gender (c)	−0.09 (0.21)			[−0.509, 0.326]		
Sport (d)	−0.06 (0.21)			[−0.482, 0.357]		
Interaction (a × c)	0.05. (0.07)			[−0.096, 0.204]		
Interaction (a × d)	0.03 (0.08)			[−0.119, 0.186]		
Interaction (b × c)	−0.02 (0.08)			[−0.146, 0.282]		
Interaction (b × d)	−0.11 (0.08)			[−0.265, 0.036]		
	**Model 1:** **Well-being**		**Model 2:** **Training readiness bias**		**Model 3:** **Competition readiness bias**	
	**B**	**95% CI**	**B**	**95% CI**	**B**	**95% CI**
COVID-19 risk (a)	−0.37 (0.12) **	[−0.617, −0.138]	0.24 (0.17)	[−0.230, 1.813]	0.24 (0.19)	[−0.124, 0.607]
Fear of COVID-19 (b)	−0.06 (0.04)	[−0.142, 0.022]	0.36 (0.06) ***	[0.249, 0.478]	0.37 (0.06) ***	[0.250, 0.500]
Safety	0.20 (0.04) ***	[0.119, 0.276]	−0.29 (0.06) ***	[−0.396, −0.176]	−0.41 (0.06) ***	[−0.528, −0.288]
Gender (c)	−0.76 (0.21) ***	[−1.169, − 0.342]	0.55 (0.29)	[−0.026, 1.130]	0.49 (0.32)	[−0.143, 1.118]
Sport setting (d)	−0.06 0(.21)	[−0.473, −0.357]	−0.23 (0.30)	[−0.814, 0.347]	0.25 (0.32)	[−0.380, 0.886]
Interaction (a × c)	0.18 (0.07) *	[0.036, 0.333]	−0.15 (0.11)	[−0.359, 0.057]	−0.08 (0.12)	[−0.308, 0.145]
Interaction (a × d)	0.05 (0.08)	[−0.105, 0.189]	0.05 (0.10)	[−0.160, 0.263]	−0.09 (0.12)	[−0.325, 0.137]
Interaction (b × c)	0.10 (0.08)	[−0.047, 0.249]	−0.06 (0.10)	[−0.265, 0.148]	−0.08 (0.11)	[−0.306, 0.144]
Interaction (b × d)	0.02 (0.08)	[−0.152, 0.015]	0.07 (0.11)	[−0.141, 0.276]	0.03 (0.12)	[−0.194, 0.261]

Note. * *p* < 0.05. ** *p* < 0.01. *** *p* < 0.001. Lowercase letters in round brackets indicate, respectively, (a) COVID-19 risk, (b) Fear of COVID-19, (c) Gender, (d) Sport.

**Table 5:** Indirect effects mediated by Safety in the hypothesized model (*N* = 619).

Predictor	Moderators’ Level	Dependent Variable	Mean Bootstrap Estimate	Percentile CI (95%)
COVID-19 risk	Men, outdoor	Well-being	0.0167 *	[−0.077, −0.012]
COVID-19 risk	Men, indoor	Well-being	0.0153 *	[−0.067, −0.007]
COVID-19 risk	Women, outdoor	Well-being	0.0161 *	[−0.065, −0.003]
COVID-19 risk	Women, indoor	Well-being	0.0128 *	[−0.053, −0.002]
COVID-19 risk	Men, outdoor	Training readiness bias	0.0270 *	[0.016, 0.123]
COVID-19 risk	Men, indoor	Training readiness bias	0.0245 *	[0.009, 0.104]
COVID-19 risk	Women, outdoor	Training readiness bias	0.0242 *	[0.003, 0.100]
COVID-19 risk	Women, indoor	Training readiness bias	0.0194 *	[0.003, 0.078]
COVID-19 risk	Men, outdoor	Competition readiness bias	0.0354 *	[0.023, 0.163]
COVID-19 risk	Men, indoor	Competition readiness bias	0.0320 *	[0.014, 0.141]
COVID-19 risk	Women, outdoor	Competition readiness bias	0.0316 *	[0.005, 0.131]
COVID-19 risk	Women, indoor	Competition readiness bias	0.0246 *	[0.003, 0.100]
Fear of COVID-19	Men, outdoor	Well-being	0.0145	[−0.004, 0.054]
Fear of COVID-19	Men, indoor	Well-being	0.0139	[−0.030, 0.026]
Fear of COVID-19	Women, outdoor	Well-being	0.0155	[−0.013, 0.049]
Fear of COVID-19	Women, indoor	Well-being	0.0118	[−0.033, 0.014]
Fear of COVID-19	Men, outdoor	Training readiness bias	0.0196	[−0.073, 0.007]
Fear of COVID-19	Men, indoor	Training readiness bias	0.0197	[−0.037, 0.044]
Fear of COVID-19	Women, outdoor	Training readiness bias	0.0212	[−0.067, 0.019]
Fear of COVID-19	Women, indoor	Training readiness bias	0.0171	[−0.020, 0.048]
Fear of COVID-19	Men, outdoor	Competition readiness bias	0.0274	[−0.099, 0.009]
Fear of COVID-19	Men, indoor	Competition readiness bias	0.0272	[−0.050, 0.059]
Fear of COVID-19	Women, outdoor	Competition readiness bias	0.0299	[−0.092, 0.027]
Fear of COVID-19	Women, indoor	Competition readiness bias	0.0236	[−0.030, 0.064]

Note. Mean bootstrap estimates are based on 5000 bootstrap samples. Significant mediation paths are indicated with *.

## Data Availability

The data that support the findings of this study are available from the corresponding author (D.D.C.), upon reasonable request.

## Supplementary Materials

The following are available online at https://www.mdpi.com/article/10.3390/ijerph19052764/s1. File S1: Effects of the COVID-19 pandemic on well-being and sport readiness of Italian team sports players: The role of perceived safety of the training environment.
