# Supplementary material for: A Moderated Mediation Analysis of the Effects of the COVID-19 Pandemic on Well-Being and Sport Readiness of Italian Team Sports Players: The Role of Perceived Safety of the Training Environment
Source: Int J Environ Res Public Health. 2022 Feb 27;19(5):2764. doi: 10.3390/ijerph19052764 (PMC8910545; doi:10.3390/ijerph19052764)
Supplement: Supplementary file 1 [file ijerph-19-02764-s001.zip › ijerph-1606303-supplementary.pdf]

Effects of the Covid-19 pandemic on well-being and sport readiness of Italian team sports players: The role of perceived safety of the training environment

Supplementary Material

Note. Only the ad hoc created scales are provided. The standardized and adapted scales described in the Manuscript can be found via references.

1. **Perceived Safety of the Training Environment Scale**

Below are some statements regarding your perception of how safe your training environment is with respect to the Covid-19 pandemic. Please, answer the following statements indicating how much you agree with them according to this scale:
0 = Completely disagree
1 = Strongly Disagree
2 = Disagree
3 = Agree
4 = Strongly agree
5 = Completely agree

1	My Club takes the necessary safety measures to minimize the chances of Covid-19 contagion.	0	1	2	3	4	5
2	I believe that my team staff respects the Covid-19 safety measures.	0	1	2	3	4	5
3	I believe that my teammates respect the Covid-19 safety measures.	0	1	2	3	4	5
4	I believe that in my Club the common areas and the training are organized to minimize the possibility of Covid-19 contagion.	0	1	2	3	4	5
5	I am satisfied with the Covid-19 safety measures of my training environment (not only related to the gym/field but also the common areas and/or the locker rooms).	0	1	2	3	4	5

2. Sport Readiness Scale

Please, reflect on how ready you feel to train and compete now and before the Covid-19 pandemic, according to this scale:
0 = Not at all ready
1 = Not very ready
2 = A bit ready
3 = Enough ready
4 = Very ready
5 = Completely

Please indicate below how much you felt/feel READY TO TRAIN:

Before the Covid-19 pandemic	0	1	2	3	4	5
From the beginning of the Covid-19 pandemic to the present day	0	1	2	3	4	5

Please indicate below how much you felt/feel READY TO COMPETE:

Before the Covid-19 pandemic	0	1	2	3	4	5
From the beginning of the Covid-19 pandemic to the present day	0	1	2	3	4	5
